# Feasibility of implementing DRG system for inpatient on PT Askes’ providers

**DOI:** 10.1186/1472-6963-12-S1-P9

**Published:** 2012-11-21

**Authors:** Novianti Br Gultom, MM APT

**Affiliations:** 1 PT Askes (Persero), Indonesia

## 

PT Askes (Persero) uses a combination of fee-for-service (FFS) and package tariff as a provider payment mechanism. Based Wouters, Annemarie et al (1999), FFS system has a high financial risk for payor. The objectives of this paper are to give a preliminary comparison between INA-CBGs (which is Indoesian DRG) and Askes’ tariff (MoH Act number: 416/Menkes/Per/II/2011), to estimate financial risks associated with the adequacy of premium if using INA-CBGs and to gain recommendation for Askes in a preparation as an Organizing Bodies of Social Security in 2014. These three objectives are answered by calculating 10 most expensive inpatient diagnoses of Askes 2011 and comparing the cost and Length Of Stay (LOS) of both tariff. Askes’ tariff doesn’t have severity level, whereas INA-CBGs has three severity levels with five different accommodation classes (III, II, I, VIP and VVIP). The study uses variety of the lowest cost (III accommodation, severity level i), the moderate cost (II accommodation, severity level ii) and the high cost (I accommodation, severity level iii).The results showed that (1) by using III accommodation with a severity level i: Askes’ tariff higher than INA-CBGs with financial risk decrease to 73%, (2) by using II accommodation with a severity level ii: Askes’ tariff still higher than INA-CBGs with financial risk about 143%, (3) by using I accommodation with a severity level iii: Askes’ tariff lower than INA-CBGs with higher financial risk, approximately 234%. Overall, Askes’ LOS is controllable compare to the ALOS of INA-CBGs. This study recommends the alternative provider payment mechanism for Askes. If Askes uses INA-CBGs with III accommodation it will minimize the financial risk. Whereas if Askes uses II and I accommodation, then the adequacy of the existing premium must be taken into account, i.e. by the estimation of financial risk. As a leading social health insurance company in Indonesia, which will have a big number of member in 2014, PT Askes has to train the staffs about DRG (costing, coding, collecting data, auditing tool, grouping, IT, etc). On considering the complexity of the DRG scheme, rather to make the new one, it is a good beginning to start with the existing DRG which is INA-CBGs from MoH, and then gradually re-calculate the cost weight and base rate and update it yearly. Nevertheless, the adequacy of the existing premium must be taken into account. Hopefully, the health care costs for the whole population of Indonesia will be more controllable in the future.

